# From Falls to Frontal Lobe Dysfunction: A Case Report on the Neurodevelopmental Impact of Pediatric Traumatic Brain Injury

**DOI:** 10.1155/crps/4751775

**Published:** 2026-09-17

**Authors:** Manuel Glauco Carbone, Alessandro Bellini, Rossella Miccichè, Beniamino Tripodi, Bruno Pacciardi, Donatella Marazziti, Icro Maremmani, Roberta Rizzato, Giulia Gastaldello, Luca Mazzetto, Claudia Tagliarini

**Affiliations:** ^1^ Division of Psychiatry, Department of Medicine and Surgery, University of Insubria, Viale Luigi Borri 57, Varese 21100, Italy, uninsubria.eu; ^2^ VP Dole Research Group, G. De Lisio Institute of Behavioral Sciences, Via di Pratale, 3, Pisa 56121, Italy, unipi.it; ^3^ Saint Camillus International University of Health Sciences, Via di Sant’Alessandro 8, Rome 00131, Italy; ^4^ World Federation for the Treatment of Opioid Dependence, Addiction Research Methods Institute, 225 Varick Street, Suite 402, New York 10014, New York, USA; ^5^ Department of Mental Health and Addictions, Division of Psychiatry, ASST Crema, Via Largo Ugo Dossena 2, Crema 26013, Italy; ^6^ Department of Clinical and Experimental Medicine, Division of Psychiatry, University of Pisa, Via Roma 57, Pisa 56100, Italy, unipi.it; ^7^ Department of Mental Health, Psychiatric Diagnosis and Treatment Service, Sant’Elia Hospital, ASP 2, Caltanissetta, Italy

**Keywords:** case report, emotional dysregulation, executive functions, frontal lobe syndrome, traumatic brain injury

## Abstract

This case report examines the presentation and management of late‐onset frontal lobe syndrome (FLS) following early‐life traumatic brain injury (TBI). It illustrates the diagnostic challenges of this condition and the rationale for an individualized pharmacological approach. The case focuses on a young adult male who sustained two head injuries in early childhood, the more severe being a frontotemporal trauma at age 5, with debilitating FLS symptoms, including marked emotional lability and executive function deficits, emerging at age 17. This delayed presentation, characteristic of late‐onset FLS, often complicates diagnosis and treatment. Prior to referral, the patient experienced significant interpersonal conflicts, strained family dynamics, and an inability to maintain employment due to his FLS symptoms. Recognizing the profound impact of his condition, a tailored pharmacological intervention strategy was implemented, focusing on mood stabilization and emotional regulation. This approach utilized mood stabilizers, specifically lithium and valproic acid, alongside the selective serotonin reuptake inhibitor (SSRI) paroxetine. Over follow‐up, the patient demonstrated gradual improvements across multiple domains. His emotional lability decreased, alongside improved interpersonal relationships, reduced family conflict, and a greater capacity for sustained occupational engagement. Additionally, he reported subjective improvements in his overall quality of life, experiencing greater emotional well‐being and increased self‐efficacy. This case highlights the importance of considering long‐term neurological consequences of TBI, even in the absence of immediate symptoms. It also illustrates the potential value of tailored pharmacological management within a broader multidisciplinary approach.

## 1. Introduction

Traumatic brain injury (TBI) is a leading cause of disability and death among children and adolescents [1]. While precise global figures remain difficult to ascertain due to inconsistencies in definitions and reporting practices, the impact of TBI is undeniable. In the United States alone, estimates from the Centers for Disease Control and Prevention for 2013 indicate approximately 2.8 million TBI‐related events, comprising about 2.5 million emergency department visits, 282,000 hospitalizations, and 56,000 deaths, a substantial portion of which involve children [2].

Pediatric TBI presents unique challenges because symptoms may evolve over time and lead to long‐term neurocognitive and behavioral sequelae [3, 4]. Even apparently mild injuries may be followed by persistent cognitive dysfunction, memory impairment, and behavioral disturbances [5]. Frontal systems are particularly vulnerable, as both direct injury and secondary processes—including neuroinflammation, altered cerebral blood flow, and disruption of ongoing neurodevelopment—may contribute to delayed clinical manifestations [6–10].

The immature brain is particularly susceptible to secondary insults, and disruption of ongoing developmental processes may lead to delayed cognitive and neuropsychiatric consequences [11, 12]. Such delayed presentations are not uncommon: in the only pediatric cohort prospectively followed into adulthood, a novel psychiatric disorder absent before the injury was still present in roughly half of individuals a mean of 24 years after a TBI requiring hospitalization, with rates of novel attention‐deficit/hyperactivity disorder (ADHD) and generalized anxiety disorder well above general‐population expectations [1]. This trajectory is not uniform across patients but has been consistently associated with greater injury severity, a pre‐injury psychiatric disorder, frontal white‐matter involvement, family dysfunction, and lower socioeconomic status [1, 13]. Longitudinal monitoring and neuroimaging follow‐up are therefore important for characterizing pediatric TBI trajectories and identifying emerging complications [14].

Pediatric TBI may disproportionately affect the frontal lobes, contributing to delayed frontal lobe syndrome (FLS) [15, 16]. FLS following pediatric TBI can manifest as impairments in executive functions (including attention and working memory), emotional regulation, and social behavior, sometimes closely resembling frontotemporal dementia (FTD) [17]. Indeed, FLS and behavioral variant FTD may share behavioral and neuroimaging features, including disinhibition, apathy, executive dysfunction, and frontotemporal atrophy, thereby complicating the differential diagnosis [18–20]. Moreover, depending on the location and extent of the injury, motor deficits, such as difficulties with motor planning and coordination, may also arise [21] (Table 1).

**Table 1:** Stages of frontal lobe syndrome development.

Stage	Clinical‐developmental description	Key mechanisms	Symptoms/manifestations
1. Initial injury	The developing brain, particularly the frontal lobes, is highly vulnerable to traumatic brain injury because of its anatomical position and ongoing maturation. Even seemingly mild injuries may disrupt critical developmental processes	Direct disruption of frontal neural networks; vulnerability related to neuroplasticity and incomplete maturation	Often subtle or clinically silent; deficits may not be immediately apparent
2. Delayed onset of symptoms	The initial trauma may trigger secondary injury cascades that emerge gradually and do not necessarily produce immediate clinical manifestations	Neuroinflammation, altered cerebral blood flow, cell death, and disruption of frontal‐lobe developmental processes	Delayed emergence of neurological, cognitive, behavioral, or psychiatric symptoms
3. Emergence of FLS	Over time, the cumulative effects of the initial injury and secondary insults may manifest as a frontal lobe syndrome	Combined effects of initial damage, long‐term neuroinflammatory processes, frontal network dysfunction, and impaired synaptic pruning	Executive dysfunction, emotional dysregulation, impulsivity, disinhibition, irritability, apathy, aggression, social inappropriateness, and difficulty adapting to change
4. Long‐term consequences	Frontal lobe syndrome may have broad functional consequences affecting academic performance, social relationships, occupational functioning, and quality of life	Persistent disruption of executive, emotional, and behavioral systems; possible contribution to long‐term neuropsychiatric vulnerability	Academic and occupational difficulties, interpersonal impairment, reduced quality of life, and increased risk of long‐term mental health problems

The present article describes the clinical presentation of a 21‐year‐old male with a history of severe frontotemporal TBI at age 5. He presented to the University Clinic of Pisa with mood instability, symptoms suggestive of obsessive–compulsive spectrum disorder (specifically, intrusive thoughts), impaired executive function, behavioral disinhibition, and impulse control issues. These symptoms followed a chronic course with progressive worsening over time. The patient was treated with a personalized pharmacological regimen based on mood stabilizers (valproic acid and lithium carbonate) and paroxetine, which led to a gradual improvement in mood, intrusive thoughts, and behavioral symptoms, with only partial improvement in executive function deficits.

## 2. Case Presentation

### 2.1. Sociodemographic Details

The patient was a 21‐year‐old unemployed male who lived with his parents and siblings. He obtained a middle school diploma but left high school after failing three consecutive years due to poor academic performance and social difficulties.

### 2.2. History of Present Illness

At age 19, he began experiencing dysphoric mood with verbal aggression, emotional lability, affective numbing, and impulsivity, without identifiable stressors. He also reported anxiety with panic attacks and agoraphobic avoidance, pathological self‐reference, suspiciousness, harm and persecution ideas, sleep disturbances, and disordered eating. He received psychiatric assessment and was treated for 2 years with several benzodiazepines and antipsychotics without significant improvement (Table 2). He then consulted a private psychiatrist who initiated valproic acid 750 mg/day and clonazepam 1 mg/day, reduced olanzapine, and discontinued other medications. Due to the lack of progress after 2 months, hospitalization was recommended.

**Table 2:** Timeline of clinical events and treatment.

Age/period	Event/clinical manifestation	Additional details
Early childhood		
Birth	Born full‐term	No complications
3 years old	Trauma 1: fall down the stairs → occipital fracture, epidural hematoma	EEGs unremarkable, hematoma resolution
3–4 years old	Psychologist: shy, good social skills, no ADHD/ASD	Reserved, rule‐abiding
5 years old	Trauma 2: road traffic accident → frontotemporal subdural hematoma, coma	Right frontotemporal subdural hematoma, parasagittal fracture, 5‐day coma. Malacic changes in frontal convexities, small hypodense spot in right frontal area. Moderate theta‐delta activity in left parietal‐occipital regions
5–10 years old	Neuropsychiatry: post‐concussive disorder (DSM‐IV/ICD‐10 framework)	Emotional lability, irritability, unmotivated mood swings, hypersensitivity to noises, frequent headaches, nocturnal enuresis
Adolescence		
11–16 years old	Satisfactory school performance, emotional stabilization	—
17 years old	Academic decline, social withdrawal begins	Decreased school attendance and reduced social interaction. Transferred schools twice unsuccessfully, resulting in three consecutive years of failing grades
Adulthood		
19 years old	Emergence of clinically significant adult psychiatric symptoms: dysphoria, anxiety with panic attacks and agoraphobic avoidance, ideas of reference, sleep disturbance, disordered eating, verbal aggression	Dysphoric mood with verbal aggression, emotional lability, affective numbing and impulsivity, anxiety with panic attacks and agoraphobic avoidance, ideas of reference, suspiciousness, persecutory ideation, ego‐dystonic harm‐related obsessions, sleep disturbances and disordered eating
19–21 years old	Followed by local psychiatric services. Treatment: benzodiazepines and antipsychotics (limited efficacy)	Treated for 2 years with various combinations of benzodiazepines (brotizolam, triazolam, flurazepam, alprazolam, lorazepam) and antipsychotics (aripiprazole, paliperidone, amisulpride, risperidone, olanzapine) without significant improvement
21 years old	Private psychiatrist: valproic acid (750 mg/day) and clonazepam (1 mg/day) initiated; olanzapine reduced; other previous medications discontinued	Lack of progress after 2 months; hospitalization recommended

During the 3 months preceding admission, low mood, heightened emotional sensitivity, irritability, and anger had persisted. Anxiety was moderate to severe, with recurrent panic attacks and agoraphobic avoidance; he reported using alcohol in an attempt to alleviate these symptoms. He also described reduced interest in socializing and previously enjoyed activities. Sleep was delayed and fragmented, with approximately three nocturnal awakenings. Eating behavior was characterized by binge‐eating episodes followed by self‐induced vomiting, with an approximately 5‐kg weight loss over the preceding month.

### 2.3. Physical Examination

Physical examination revealed no significant abnormalities. His body mass index (BMI) was 17.2.

### 2.4. Mental Status Examination

At the examination, the patient was alert and fully oriented, with appropriate appearance and hygiene. A mild distal tremor was noted. He was cooperative but had difficulty inhibiting verbal responses, with occasional verbal outbursts. Speech was spontaneous, coherent, and normally paced. Mood was dysphoric and irritable, while affect was constricted and tense. He reported prominent anxiety. Thought form was linear and goal‐directed.

Thought content was characterized by ideas of reference, namely, the feeling of being constantly watched and monitored, and by persecutory ideation centered on the belief that others were plotting against him. He also reported frequent, ego‐dystonic intrusive thoughts of harming others, experienced as distressing and against his will, with a marked fear of acting upon them (impulse‐phobia), “I’m afraid of killing someone… I’m afraid of attacking people from behind… I get the urge to do it….” These were consistent with aggressive, harm‐related obsessions rather than genuine intent or planning, and he retained partial but overall fair insight into their intrusive and irrational nature. No dysmorphophobic ideas were elicited. Cognitively, he was alert, with mild but not disabling distractibility that occasionally required redirection to the task; abstraction and cognitive flexibility were preserved but limited, foreshadowing the executive difficulties subsequently confirmed on formal neuropsychological testing (Section 2.9).

### 2.5. Psychiatric Disorder History

The individual described in the case was born full‐term without complications. At age 3, he sustained a head trauma from a fall, resulting in an occipital fracture and epidural hematoma. Electroencephalograms (EEGs) during hospitalization were unremarkable, and a follow‐up computed tomography (CT) scan showed hematoma resolution. He received follow‐up with a child psychologist for 6 months, who described him as shy, reserved, and rule‐abiding, with good social skills and no signs of ADHD or autism.

At age 5, he experienced a second head trauma in a street accident, resulting in a right frontotemporal subdural hematoma, parasagittal fracture, and 5 days of coma, with subsequent neuroimaging and EEG abnormalities summarized in Table 2. Following the car accident, a neuropsychiatric consultation highlighted emotional lability, irritability, unmotivated mood swings, hypersensitivity to noises, frequent headaches, and nocturnal enuresis. Notably, these manifestations represented a qualitative change from his pre‐injury profile rather than an exacerbation of pre‐existing traits: prior to this second trauma, he had been described as shy, reserved, and rule‐abiding, with good social skills and no emotional‐behavioral dysregulation. While his baseline introverted temperament persisted, the affective lability, irritability, and neurovegetative symptoms that emerged after the accident constituted a new symptomatic domain temporally linked to the frontotemporal injury. The clinician diagnosed post‐concussive disorder, the diagnostic label available within the DSM‐IV and ICD‐10 frameworks in use at the time. Neuropsychological testing performed during the subsequent childhood follow‐up documented an overall age‐appropriate cognitive profile (full‐scale IQ in the average range) with only mild executive weaknesses (Box 1). It should be noted that the DSM‐5 no longer recognizes post‐concussive disorder as a standalone entity; under current nosology, the persistent neurocognitive and behavioral sequelae described here would be conceptualized within the framework of mild or major neurocognitive disorder due to TBI [22]. After this event, the patient was managed by infancy neuropsychiatry for emotional lability, irritability, and other symptoms. The available records indicate a course of supportive psychotherapy and psychoeducation for the child and family; no structured cognitive or neuropsychological rehabilitation program was documented. This support led to improvement and stabilization, with satisfactory school performance until age 16.

**Box 1. The Wechsler Intelligence Scale for Children and the behavior rating inventory of executive function (BRIEF) reports.**
The Wechsler Intelligence Scale for Children–Fifth Edition (WISC‐V) and the BRIEF reports are provided later in this study.
At the WISC‐V, A.S. obtained a full‐scale IQ score within the average range (100), indicating overall age‐appropriate cognitive abilities. His strengths appear to lie in verbal comprehension, as evidenced by his high score on Similarities, suggesting strong verbal reasoning and language skills. While his visual–spatial and fluid reasoning abilities are also within the average range, his performance on tasks requiring working memory and attention was somewhat impacted by his tendency for distractibility. This was particularly evident during Digit Span, where he had difficulty holding information in mind while simultaneously processing new information. The patient’s performance on tasks requiring executive functions, such as planning, organization, and inhibition, was also slightly lower than his other cognitive abilities, suggesting a mild area of relative weakness.
The patient’s performance on the BRIEF indicates average overall executive functioning. However, his scores on the Inhibit and Plan/Organize scales are mildly elevated, suggesting some difficulties with these specific areas relative to his cognitive abilities.
Specific observations, as follows:
  • Mild distractibility: While the patient’s overall score on the monitor scale is within the average range, his parent/teacher reported some mild difficulties with sustaining attention, particularly during lengthy tasks and classroom activities.
  • Planning and organization: The patient may require minor additional support with planning and organizing tasks. This may manifest as difficulty finishing homework and following multistep instructions.
  • Inhibition: The mildly elevated score on the Inhibit scale suggests that the patient may have some difficulty controlling impulses or stopping himself from doing something he should not (difficulty waiting his turn).
  • Strengths: The patient demonstrates age‐appropriate skills in initiating tasks, appropriate management of frustration, demonstrated empathy, and good attention to details.

From the age of 17, the patient experienced a marked decline in academic performance, evidenced by decreased school attendance and reduced social interaction. Despite consistently maintaining appropriate conduct and lacking any aggressive or disruptive behaviors, his academic struggles persisted. Transferring schools twice in an attempt to improve his situation proved unsuccessful, resulting in three consecutive years of failing grades.

A detailed overview of the clinical history is presented in Table 2.

### 2.6. Substance Use Disorder History

The reported daily use of approximately 1 L of beer for 1 year, along with smoking 10 cigarettes/day and consuming three coffees/day.

### 2.7. Family History

His family comprised his father and mother, who were married at the time of evaluation, and three siblings (two of them were twins), all were in good health. It was reported that the father suffered from anxiety symptoms, never treated by a psychiatrist or a psychologist, and a cousin was reported to suffer from an eating disorder, not further specified.

### 2.8. Investigations

During the hospitalization, magnetic resonance imaging (MRI) and EEG were performed.

The MRI revealed a volumetric asymmetry between the frontal lobes, with the right frontal lobe appearing smaller than the left. Frontotemporal cortical atrophy is evident, more pronounced on the right, characterized by sulcal widening and lateral ventricular enlargement, consistent with the observed atrophy. A reduced thickness of the right superior longitudinal fasciculus was observed, particularly in the segment projecting to the right frontal regions. Overall, these findings suggest a chronic process affecting the right frontal lobe.

The EEG demonstrated abnormal activity characterized by a reduction in the normal alpha (8–12 Hz) activity, which is replaced by excessive theta (4–7 Hz) activity, predominantly over the right frontal region (Fp2 and F4). Intermittent bursts of delta (1–3 Hz) activity were observed, primarily localized to the right frontal region, consistent with frontal intermittent rhythmic delta activity (FIRDA). These findings were suggestive of cortical dysfunction localized to the right frontal lobe.

### 2.9. Assessments

Executive function and cognitive assessments were performed, including the Wisconsin card sorting test (WCST) and the Tower of London test (Box 2). Both assessments revealed significant impairments in the executive functioning. Compared with the essentially age‐appropriate cognitive profile documented in childhood (average full‐scale IQ with only mild executive weaknesses; Box 1), the present assessment revealed significant executive dysfunction (Box 2), consistent with a decline over time rather than a static, longstanding deficit.

**Box 2. WCST and Tower of London reports.**
During the execution of both tests, the patient exhibited signs of frustration and impatience, particularly when faced with changing rules and more complex problems. He displayed difficulty inhibiting previously successful strategies, often perseverating on a sorting rule even after receiving negative feedback. He displayed a tendency to rush through the task, often making impulsive moves without adequate planning. His attention wandered at times, requiring redirection to the task.
The patient’s performance on the WCST is suggestive of impairments in executive functioning, particularly in the following areas:
  • Set shifting/cognitive flexibility: difficulty adapting to changing rules and generating new problem‐solving strategies, as evidenced by the high number of perseverative errors and low categories completed.
  • Inhibition: difficulty suppressing prepotent responses and inhibiting impulsive behaviors, contributing to the perseverative errors observed.
  • Working memory: may have contributed to difficulty maintaining the current sorting rule in mind, potentially impacting his ability to shift sets effectively.
On the other hand, the patient’s average performance on nonperseverative errors suggests intact basic card‐sorting abilities and rule comprehension. The patient’s performance on the WCST, in conjunction with his clinical presentation, suggests difficulties with executive functioning, particularly in the areas of set‐shifting, inhibition, and working memory. These difficulties may be contributing to his challenges with academic performance and behavioral problems.
The patient’s performance on the Tower of London test suggests difficulties in the following areas:
  • Planning: difficulty formulating and executing efficient problem‐solving strategies, as evidenced by the increased time taken and lower number of problems solved in the minimum moves.
  • Inhibition: difficulty suppressing impulsive actions and thinking before acting, contributing to the higher number of rule violations and inefficient problem‐solving.
  • Working memory: may have contributed to difficulty holding the problem requirements and potential solutions in mind, impacting his ability to plan effectively.
The patient’s ability to eventually solve the problems, even if not in the minimum moves, suggests intact basic problem‐solving skills and rule comprehension, although his approach was marked by trial‐and‐error rather than a systematic strategy.

### 2.10. Diagnosis

The diagnosis of FLS has been established based on the following criteria:

• Clinical evaluation: cognitive changes (memory, attention, and executive functions) and psychopathological expressivity (behavioral changes with dysphoric mood, emotional lability, affective numbing, lack of impulse control, sleep, and eating disturbances)
• Neurological assessment: clinical evaluation conducted by a neurologist specializing in neurodegenerative and neurodevelopmental disorders, supported by clinical history, neuroimaging findings, and psychodiagnostic testing.
• Diagnostic tests: evaluation of brain activity (EEG) and structural brain imaging (MRI), and administration of neuropsychological testing (WCST and Tower of London).

#### 2.10.1. Differential Diagnosis

Diagnostic assessment was complicated by the long interval between the childhood injury and the clinically significant adult presentation and the unavailability of the original childhood neuroimaging and EEG recordings. Alternative diagnoses were weighed before adopting FLS. Bipolar and schizophrenia spectrum disorders were considered. However, the available history did not document a sustained syndromic manic episode or an autonomous psychotic syndrome. Suspiciousness and ideas of reference fluctuated with anxiety and panic symptoms, while the broader presentation followed a documented post‐TBI developmental trajectory and was accompanied by focal right‐frontal MRI and EEG abnormalities and acquired executive dysfunction. Taken together, these features favored a secondary neuropsychiatric origin. A primary personality disorder was considered, but the acquired emergence of affective lability and impulsivity against a well‐adjusted premorbid profile indicated personality change due to a general medical condition [23]. The ego‐dystonic, harm‐related intrusive thoughts were interpreted as obsessive–compulsive symptoms secondary to orbitofronto‐striato‐thalamo‐cortical dysfunction rather than primary obsessive–compulsive disorder (OCD) [24]. Finally, FLS is used here as a descriptive construct complementary to, rather than competing with, the DSM‐5 category of mild or major neurocognitive disorder due to TBI [22]; behavioral‐variant FTD, which FLS may phenocopy, was deemed unlikely given the young age and the congruent history of focal traumatic injury, though longitudinal monitoring remains warranted.

### 2.11. Treatment Details and Course in the Hospital

The pharmacological strategy was guided by the patient’s predominant symptom domains and by his documented treatment resistance. Valproic acid, already initiated before admission, was uptitrated toward an optimal dose (from 750–1500 mg/day) to target mood lability, dysphoria, and behavioral disinhibition; its anticonvulsant, membrane‐stabilizing action provided an additional patient‐specific rationale in light of the focal right‐frontal electrophysiological abnormalities (frontal slowing and FIRDA). Rather than pursuing a slower sequential optimization, lithium carbonate was introduced concurrently, adopting a dual mood‐stabilizer strategy from the outset, given the severity of the presentation and the prior nonresponse to benzodiazepines and multiple antipsychotics. Lithium was selected specifically to address the residual mood instability and the prominent aggressive ideation and impulsivity, for which its anti‐aggressive and anti‐impulsive effects are well established, and for its neurotrophic and neuroprotective properties, of particular relevance in the context of TBI‐related neuroprogression (refer to Section 3). Paroxetine was added to target anxiety, panic‐agoraphobic symptoms, and the ego‐dystonic intrusive thoughts, while low‐dose olanzapine was maintained transiently for suspiciousness and ideas of reference and subsequently withdrawn once stabilization was achieved, consistent with the aim of minimizing antipsychotic and benzodiazepine exposure.

During hospitalization, paroxetine was titrated to 20 mg/day and lithium carbonate to 600 mg/day. The patient experienced transient accelerated thinking. Over the following days, intrusive thoughts and tension decreased, and sleep and appetite improved. Neurological consultation revealed no new abnormalities. The patient was discharged on valproic acid 1500 mg/day, lithium carbonate 600 mg/day, clonazepam 1.5 mg/day, paroxetine 20 mg/day, and olanzapine 2.5 mg/day.

One‐month follow‐up showed reduced aggressive ideation and anxiety. Ideas of reference, avoidance, and persecutory ideation persisted. The patient reported a weight gain. Olanzapine was discontinued, clonazepam was tapered, and lithium carbonate was increased to 750 mg/day.

Three‐month follow‐up showed further improvement, near‐absence of ideas of reference, sustained remission of the intrusive, harm‐related obsessive thoughts, and return to social activities. Since returning home, the patient has reportedly reduced his alcohol consumption, which he had previously used as a maladaptive strategy to cope with anxiety, panic, and agoraphobic symptoms.

Six‐month follow‐up confirmed improvements; attention and distractibility improved, but executive functions (such as work organization, time management, medium‐ to long‐term planning, and cognitive flexibility) remained impaired. The pharmacological regimen was therefore maintained unchanged.

One‐year follow‐up showed good stability, appropriate behavior, and employment. The patient reported having commenced continuous work as a laborer alongside his father a little over 2 months prior—an activity he had never undertaken before. The occupational placement began within a family‐supported setting under his father’s supervision and progressively evolved toward greater autonomy as the patient acquired competence in his tasks, although a degree of paternal supervision remained in place. This trajectory represents a meaningful functional gain relative to his previous inability to sustain any occupation, while the initially supported and gradually autonomizing nature of the setting should be considered when interpreting the extent of his functional recovery. The blood levels of valproic acid and lithium were within the normal range, and routine blood tests were normal. Medication adherence was reported as satisfactory by the patient and his family. The treatment plan was likewise unchanged.

A detailed overview of the timeline of symptoms and response to treatment is presented in Table 3.

**Table 3:** Chronological progression of symptoms and treatment outcomes.

Time point	Setting	Event/clinical manifestation
21 years old	Hospitalization	Paroxetine and lithium carbonate initiated (paroxetine 20 mg/day, lithium carbonate 600 mg/day, valproic acid 1500 mg/day). Olanzapine reduced (2.5 mg/day at discharge), clonazepam tapered (1.5 mg/day). Transient accelerated thinking
21 (+1 month)	Follow‐up	Reduced aggressive ideation and anxiety. Ideas of reference, avoidance, and persecutory ideation persisted. Weight gain reported. Olanzapine discontinued, clonazepam tapered, lithium carbonate increased to 750 mg/day
21 (+3 months)	Follow‐up	Further improvement, near‐absence of ideas of reference, sustained remission of intrusive obsessive thoughts, return to social activities. Reportedly reduced alcohol consumption
21 (+6 months)	Follow‐up	Confirmed improvements; attention and distractibility improved, but executive functions (work organization, time management, medium‐ to long‐term planning, and cognitive flexibility) remained impaired. Pharmacological regimen maintained
22 years old	Follow‐up	Good stability: employment as a laborer alongside his father and appropriate behavior. Valproic acid and lithium blood levels within normal range. Routine blood test results were within normal limits. Pharmacological treatment plan remained unchanged

## 3. Discussion

This case highlights the delayed neuropsychiatric consequences of pediatric TBI, particularly when frontal systems are involved. It supports the view that childhood TBI may act not only as an acute injury but also as a chronic vulnerability, with evolving clinical manifestations over time [25–28].

Regarding the symptomatology, while the clinical presentation strongly suggests a neurodegenerative cascade triggered by early trauma, it is prudent to consider other differential etiologies. The observation that alternative explanations could include primary psychiatric conditions warrants a closer examination. A bipolar spectrum disorder might be considered given the mood instability, impulsivity, and transient accelerated thinking and a schizophrenia spectrum disorder given the suspiciousness, ideas of reference, and persecutory ideation. Several features favored a secondary neuropsychiatric formulation: the available history did not demonstrate a sustained manic episode or an autonomous psychotic syndrome; suspiciousness and ideas of reference fluctuated with anxiety and panic symptoms; the presentation followed a documented behavioral and developmental change after TBI; and it was accompanied by acquired executive dysfunction and focal right‐frontal structural and electrophysiological abnormalities. None of these features is independently diagnostic, and a case report cannot exclude a comorbid primary psychiatric disorder. His neuroimaging findings—cortical atrophy, lobar asymmetry, and neuronal loss—correspond closely with known sequelae of childhood TBI [29, 30]. The temporal and spatial correspondence between the initial injury, these neuroanatomical alterations, and the progression of symptoms is consistent with—though, in a single case, cannot by itself establish—a predominant contribution of trauma‐related neurodegeneration to his clinical profile, while acknowledging that a case report cannot exclude contributory or coincidental factors [27, 31].

The contribution of personality pathology also deserves consideration, particularly in light of the impulsivity and emotional dysregulation that characterized much of the patient’s course. These traits, however, did not reflect a primary, lifelong personality disorder: prior to the age‐5 frontotemporal injury, the patient had been described as shy, reserved, rule‐abiding, and socially well‐adjusted, with affective lability, irritability, and impulsivity emerging only thereafter. This acquired trajectory, together with the frontal neuroanatomical substrate, is most consistent with personality change due to a general medical condition rather than a primary personality disorder such as borderline personality disorder [23]. This distinction is clinically important as it reframes the impulsivity and dysregulation as expressions of the underlying frontal syndrome rather than as an independent, trait‐based comorbidity.

These considerations raise the broader question of how persistent post‐TBI symptoms should be classified. The DSM‐5 no longer recognizes post‐concussive disorder as an independent entity and instead situates the enduring cognitive and behavioral consequences of TBI within mild or major neurocognitive disorder due to TBI, defined by objective cognitive decline and its functional impact [22]. FLS, by contrast, is not a formal diagnostic category but a descriptive, syndromic construct denoting the dysexecutive, disinhibited, and affectively dysregulated phenotype arising from frontal dysfunction. In the present case, the two frameworks are complementary rather than mutually exclusive: FLS captures the phenomenological and neuroanatomical character of the presentation, whereas mild/major neurocognitive disorder due to TBI provides its formal nosological placement. We, therefore, adopt FLS as a clinically descriptive label while recognizing that, within the current nosology, the patient’s presentation corresponds to a neurocognitive disorder due to TBI with prominent behavioral disturbance.

A primary neurodegenerative disorder such as behavioral‐variant FTD, which FLS may phenocopy, was considered less likely given the young age and the congruent history of focal traumatic injury, although the possibility underscores the need for continued longitudinal surveillance.

These observations reinforce the importance of structured long‐term surveillance after pediatric TBI, including periodic neuropsychological reassessment, neuroimaging when clinically indicated, and monitoring for emerging cognitive or behavioral decline [28, 32, 33].

This case also illustrates a clinically relevant aspect of pharmacotherapy: lithium and valproic acid were employed not merely as mood stabilizers but for their neuroprotective and neurotrophic properties, which are of particular interest in a disorder conceptualized as trauma‐induced neuroprogression. Beyond their established mood‐stabilizing actions, both agents exert anti‐inflammatory and neurotrophic effects—such as lithium’s promotion of BDNF and attenuation of neuroinflammation and valproate’s modulation of gene expression through histone deacetylase inhibition—that may, in principle, favorably modify the disease course in the context of TBI [34–40].

The addition of paroxetine, a selective serotonin reuptake inhibitor (SSRI), proved effective in managing the patient’s anxiety, panic‐agoraphobic symptoms, and ego‐dystonic intrusive thoughts, illustrating a key feature of this presentation: the tight coupling between affective, anxiety‐related, and behavioral symptoms [41]. Anxiety was not a peripheral complaint but a core driver of the clinical picture. Frontal dysfunction impairs the regulation of emotional and autonomic responses, plausibly contributing to the patient’s heightened anxiety and recurrent panic attacks, which in turn fostered agoraphobic avoidance in a self‐reinforcing cycle [42]. In this patient, the intrusive thoughts were ego‐dystonic and distressing, centered on fears of harming others against his will, a phenomenology consistent with the aggressive/harm obsessions of the obsessive–compulsive spectrum rather than with disinhibited impulsivity alone; such post‐TBI obsessive–compulsive symptoms mirror idiopathic OCD and have been linked to orbitofronto‐striato‐thalamo‐cortical dysfunction, a network directly compromised by this patient’s right frontal pathology [24, 43]. The suspiciousness and ideas of reference are best described as subthreshold paranoid phenomena that fluctuated with anxiety and panic symptoms and did not form an autonomous psychotic syndrome [44–46]. By enhancing serotonergic signaling, paroxetine likely acted on this anxiety‐driven core, reducing panic and intrusive symptoms; the accompanying decrease in alcohol use is consistent with alcohol having served as a maladaptive coping strategy for anxiety and panic symptoms [47].

This case underscores the crucial importance of individualized medication management. The discontinuation of antipsychotics and benzodiazepines, drugs that may impair executive functions and cognitive processes, was associated with stabilized cognitive functioning and improved social engagement in this patient. Evidence from mechanistic and clinical work suggests that antipsychotics can contribute to cognitive impairment and executive dysfunction, particularly through their effects on frontostriatal and dopaminergic systems [48–50]. Similarly, both longitudinal studies and meta‐analytic data indicate that long‐term benzodiazepine use is associated with subtle but clinically relevant decrements in memory, attention, and global cognition, especially in older or vulnerable individuals [51–53]. In this context, the temporal association between deprescribing and cognitive/social improvement in our case aligns with this literature and highlights the need for ongoing monitoring and treatment adjustments tailored to the patient’s evolving clinical picture.

The pharmacological adjustments may have contributed to clinical stability, with improvements in cognitive function and social engagement, emphasizing the interconnectedness of these domains. Better emotional regulation and reduced anxiety likely facilitated his engagement in cognitive tasks and executive function recovery, highlighting the importance of addressing emotional and behavioral factors for cognitive rehabilitation.

Looking ahead, maintaining stability through treatment adherence and regular monitoring is vital. Implementing targeted compensatory strategies tailored to his cognitive challenges will further support his autonomy.

Although the patient improved clinically, early‐life TBI remains associated with increased long‐term neuropsychiatric and neurodegenerative vulnerability [54–60]. Continued neurological monitoring, cognitive follow‐up, lifestyle interventions, and compensatory executive strategies are therefore warranted to support long‐term functioning and detect any emerging decline.

A principal strength of this report is the integration of longitudinal developmental and clinical records with current neuropsychological testing, structural neuroimaging, electrophysiological findings, and 1‐year functional follow‐up. This multimodal reconstruction allowed the adult presentation to be interpreted within the context of the patient’s documented neurodevelopmental trajectory.

Several limitations should be acknowledged. As a single case report, these observations are hypothesis‐generating and cannot establish causality or be generalized. The neuroimaging and EEG studies performed at the time of the childhood injuries were obtained at another institution many years earlier and could not be retrieved, precluding a direct longitudinal comparison of images and waveforms; only the original radiological and EEG reports were available. Sleep was characterized clinically rather than by polysomnography, and the EEG described represents a standard waking recording without sleep‐stage data. Finally, part of the early developmental and treatment history was necessarily reconstructed from retrospective records.

## 4. Conclusion

This case report highlights the complex and often prolonged challenges of late‐onset FLS after early‐life TBI. The patient’s experience emphasizes the need for personalized, holistic treatment approaches that go beyond addressing the immediate symptoms.

His positive clinical response suggests that individualized medication management may have contributed to the observed improvement. The use of mood stabilizers, such as lithium and valproic acid, combined with the SSRI paroxetine, may have helped manage his emotional lability and impulsivity, thereby contributing to his overall well‐being. However, this improvement should be interpreted within a broader multifactorial context, including psychosocial stabilization, occupational engagement, reduced alcohol consumption, and environmental changes. Similarly, minimizing medications such as antipsychotics and benzodiazepines, which may contribute to cognitive and functional difficulties in some patients, may also have been beneficial, highlighting the importance of ongoing medication review tailored to the patient’s clinical history.

Despite these successes, the patient’s early TBI poses a long‐term risk for neuroinflammatory and neurodegenerative conditions. This requires a proactive approach, with regular neurological assessments and lifestyle modifications to support brain health. Early detection of any decline is vital for timely intervention and better long‐term outcomes.

Finally, this case underscores the importance of open communication and a strong therapeutic alliance among the patient, their support network, and the healthcare team.

Recognizing both the potential for recovery and the risks linked to early TBI, a collaborative, patient‐centered approach may help optimize long‐term outcomes.

## Author Contributions

**Manuel Glauco Carbone**: conceptualization, formal analysis, writing – original draft. **Rossella Miccichè and Luca Mazzetto**: literature search. **Giulia Gastaldello and Beniamino Tripodi**: writing – review and editing. **Alessandro Bellini and Claudia Tagliarini**: supervision, writing – review and editing. **Roberta Rizzato**: literature search, writing – original draft. **Bruno Pacciardi, Donatella Marazziti, and Icro Maremmani**: supervision, writing – review and editing, writing – original draft.

## Funding

This research did not receive any specific grant from funding agencies in the public, commercial, or not‐for‐profit sectors. Open access publishing facilitated by Universita degli Studi dellInsubria, as part of the Wiley ‐ CRUI‐CARE agreement.

## Disclosure

All AI‐assisted edits were reviewed and verified by the authors for accuracy.

## Consent

According to institutional and national regulations, single‐patient case reports that describe routine clinical practice and contain anonymized data do not require approval from an Ethics Committee. Written informed consent was obtained from the patient for the publication of this case report.

## Conflicts of Interest

The authors do not have an affiliation with or financial interest in any organization that might pose a conflict of interest.

## Patient Perspective

During follow‐up, the patient reported subjective improvement in emotional well‐being, social functioning, and self‐efficacy. He considered his return to work and greater autonomy to be relevant improvements in his daily life.

## Data Availability

The data that support the findings of this study are available from the corresponding author upon reasonable request.
